# Mesoscale Modeling of Agglomeration of Molecular Bottlebrushes: Focus on Conformations and Clustering Criteria

**DOI:** 10.3390/polym14122339

**Published:** 2022-06-09

**Authors:** Sidong Tu, Chandan K. Choudhury, Michaela Giltner, Igor Luzinov, Olga Kuksenok

**Affiliations:** Department of Materials Science and Engineering, Clemson University, Clemson, SC 29634, USA; sidongt@clemson.edu (S.T.); chandan.k.choudhury@gmail.com (C.K.C.); michaela.giltner@slu.edu (M.G.); luzinov@clemson.edu (I.L.)

**Keywords:** molecular bottlebrush, dissipative particle dynamics, self-assembly, aggregation number

## Abstract

Using dissipative particle dynamics, we characterize dynamics of aggregation of molecular bottlebrushes in solvents of various qualities by tracking the number of clusters, the size of the largest cluster, and an average aggregation number. We focus on a low volume fraction of bottlebrushes in a range of solvents and probe three different cutoff criteria to identify bottlebrushes belonging to the same cluster. We demonstrate that the cutoff criteria which depend on both the coordination number and the length of the side chain allows one to correlate the agglomeration status with the structural characteristics of bottlebrushes in solvents of various qualities. We characterize conformational changes of the bottlebrush within the agglomerates with respect to those of an isolated bottlebrush in the same solvents. The characterization of bottlebrush conformations within the agglomerates is an important step in understanding the relationship between the bottlebrush architecture and material properties. An analysis of three distinct cutoff criteria to identify bottlebrushes belonging to the same cluster introduces a framework to identify both short-lived transient and long-lived agglomerates; the same approach could be further extended to characterize agglomerates of various macromolecules with complex architectures beyond the specific bottlebrush architecture considered herein.

## 1. Introduction

Molecular bottlebrushes (MBBs) are branched macromolecules with the side chains densely grafted to the linear backbone [1,2,3,4,5,6]. The structural characteristics of the bottlebrushes can be tailored during their synthesis by regulating their grafting density, degrees of polymerization of the side chains and the backbone, and chemical composition [6,7,8,9]. Due to their unique properties, MBBs can find their use in various applications, from electronic and photonic materials [10,11,12] to strain-adaptive stiffening materials [2,7] and drug delivery applications [13,14]. High grafting density leads to the pronounced effects of steric repulsions, which results in the stretched conformations of a backbone and side chains compared with the linear chain conformations. The extent of stretching, as well as equilibrium conformation of the MBB, strongly depends on its architecture, chemical nature of the moieties within the backbone and side chains, solvent quality, and concentration of MBBs within the solvent [1,6,15,16]. Relatively high backbone tension can develop depending on bottlebrush conformation [17,18]; this tension can be further amplified upon bottlebrush adsorption onto the interface [19,20,21]. 

For a chosen architecture and chemical nature of a bottlebrush, its conformation can be tuned either by regulating the solvent quality or by regulating the concentration of bottlebrushes in solution. The phase separation and agglomeration processes in blends and solutions incorporating MBBs—as well as migration of bottlebrushes to the interfaces and surfaces in multi-phase systems—introduce further complexity, so that the resulting behavior depends on the interplay between the contributions from the components with various affinities [22,23,24,25]. The self-assembly process can also be tailored by external stimuli, such as temperature, pH, ionic strength, and external fields [2,26,27,28,29]. For example, the conformation of the bottlebrush with side chains exhibiting lower critical solution temperature (LCST) behavior depends strongly on the architectural properties of the bottlebrush as well as on temperature [30,31,32]. At low temperatures, the side chains are hydrophilic, hence the bottlebrush attains cylindrical morphology provided that the backbones are significantly longer than the side chains [30]. An increase in temperature results in a pronounced decrease in the bottlebrush radius of gyration and in cylinder-to-globule transition at sufficiently high temperatures [30,32]. Furthermore, a two-step conformational change with an increase in temperature was reported for polymers with molecular architecture intermediate between a star-like and a cylindrical bottlebrush: an increase in temperature first resulted in stretching and only then in collapse of the macromolecules at sufficiently high temperatures [33]. The chemical nature of the end groups along with the length of the side chain also significantly affects phase behavior and interfacial properties of the bottlebrushes [31,34]. Varying the architecture of the stimuli-responsive side chains by choosing either random or block copolymers further allows one to tailor bottlebrush conformation and aggregation behavior [35]. Recent experiments showed that the bottlebrushes within aggregates formed at high temperatures (above LCST) are either loosely packed if the side chains are random copolymers or strongly interpenetrate if the side chains are block copolymers [35]. 

Of particular interest is using a relatively low fraction of specifically designed bottlebrushes to controllably tailor bulk and surface properties of various systems. Bottlebrushes can be effectively used as compatibilizers to control morphologies and characteristic domain sizes of immiscible polymer blends; specifically, MBBs were shown to encapsulate one of the components of the polymer blend, thereby suppressing coarsening at elevated temperatures [36,37]. Bottlebrushes were also shown to encapsulate proteins such as lysozyme, preventing its denaturation at high temperatures [38]. Tailoring architecture and composition of MBBs allows one to form micelles with controllable sizes [14,39] and imparts the capability to carry drugs with varied hydrophobicity [40,41]. 

In our recent study [42], oleophobic perfluoropolyether (PFPE) methacrylate molecular bottlebrush (PFM) was synthesized and used as an additive to commodity thermoplastics (nylon 6 and poly(methyl methacrylate)). Our studies showed that an addition of only 1% to 5% of the PFM bottlebrush additives imparts high water and oil repellency to the host thermoplastic matrix. While similar oleophobic properties can be achieved via utilizing long-chain perfluoroalkyl substances (LCPFAS), these materials have currently been phased out due to their negative environmental impact [43,44,45]. PFM bottlebrush additives introduce a feasible and potentially safer alternative to LCPFAS due to the lower toxicity and high oxidative and thermal stability of perfluoropolyethers [46,47,48,49]. We showed that the observed high oil repellency of the surfaces is due to the migration of PFM bottlebrush additives to the thermoplastics surfaces due to the mismatch in the affinity between the densely grafted side chains and a host polymer [42]. Specifically, we showed that first the bottlebrushes aggregate into the clusters with various number of brushes depending on the affinity between the moieties comprising the backbone/side chain and the host matrix. Upon approaching the interface, the bottlebrush clusters spread over the interface exposing highly oleophobic side chains [42]. 

The characterization of bottlebrush conformations is an important step in understanding the relationship between the bottlebrush architecture and material properties [50]. A number of techniques is available for the direct visualization of the conformation of MBBs in experiments, including atomic force microscopy [7,51], transmission electron microscopy, small-angle neutron scattering [52], and single-molecule localization microscopy [53]. However, due to the limited resolution of available experimental techniques [53,54], it remains challenging to distinguish the bottlebrushes within clusters and characterize their conformations. Computational modeling allows one to understand and predict bottlebrush conformations under various conditions [5,22,23,51,52,55,56,57,58,59,60,61,62,63,64]; current modeling efforts focusing on characterizing self-assembly of bottlebrushes are surveyed in a recent review by Mohmmadi et al. [65]. 

Of particular relevance to the current work are recent studies characterizing conformations of MBBs in solvents of various quality. Recent integrated computational and experimental study quantified the effects of the backbone and side chain lengths on the conformations of bottlebrushes in a good solvent for a range of architectural parameters of bottlebrushes [66]. Specifically, the variation in the backbone molecular weight for the fixed length of the side chain was shown to significantly affect the resulting MBB conformation, ranging from a star-like structure for a short backbone, to an extended rod, and then to a coiled chain with an increase in backbone length; importantly, these changes were reflected in intrinsic viscosity measured experimentally [66]. Furthermore, the decrease in solvent quality from good to poor solvent for a fixed bottlebrush architecture was shown to induce transitions from the coil regime to the dense collapsed state [23,55,57,60]. This transition is dependent upon the architectural parameters, such as the length of the side chain and grafting density [55,57]. In addition, the conformation of the bottlebrush also reflects variations in their concentration with scaling relations affected by the screening of excluded volume interactions [58,59]. 

For multiple bottlebrushes with various affinities between the backbone and solvent, the bottlebrushes agglomerate into clusters of various shapes—including spherical, cylindrical, or necklace-type structures—depending on the side chain grafting densities and chemical nature of the monomers [67]. For bottlebrush block copolymer with two-component side chains, the phase separation behavior in solutions was quantified with respect to the arrangement and relative fraction of the two components [22,56,61]. Recently, Gumerov and Potemkin [23] utilized dissipative particle dynamics (DPD) simulations to characterize conformations of a single MBB with responsive inner blocks of the side chains, as well as self-assembly of these MBBs, in solvents of various quality. They demonstrated that MBBs form continuous or branched aggregates or cylindrical micelles depending upon solvent quality and grafting density; an increase in grafting density was shown to change the aggregate shape and required a lower solvent quality to induce an aggregation of the MBBs [23]. Chang et al. [68] demonstrated a transition of micellar aggregates into unilamellar vesicles and then to “onion-like” multilayered vesicles with the decrease in the affinities between the side chain and solvent. This morphology was observed for the relatively short MBB molecules [68,69] and hyperbranched multi-arm copolymers [70,71]. 

Herein, we focus on characterizing the effects of the solvent quality on the average size of agglomerates in the system with low volume fraction of bottlebrushes in solvents of various quality with respect to the side chains. We characterize the changes in the conformation of the bottlebrush within the agglomerate with respect to that in the limit of infinite dilution (single bottlebrush). In what follows, we first introduce our modeling approach and the choice of model parameters; this choice is informed by our prior experimental results [42] providing experimentally relevant architectural characteristics of the bottlebrush. We then characterize radius of gyration, shape anisotropy, and an acylindricity of a single bottlebrush in solvents of various quality and compare these characteristics with the respective values calculated during the agglomeration process. We track the agglomeration process by tracking the time evolution of cluster distribution, the size of the largest cluster, and an average aggregation number. We probe three different cutoff criteria to identify whether two bottlebrushes belong to the same cluster. The results below show that the cutoff criterion which depends on both the coordination number and the length of the side chain allows one to correlate the agglomeration status with the structural characteristics of the bottlebrushes in solvents of various qualities. 

## 2. Methods

We use the dissipative particle dynamics (DPD) approach [72,73,74,75] to model the time evolution and self-assembly in bottlebrush-solvent systems. DPD is a computationally efficient mesoscale approach that has been utilized to model a broad variety of polymer systems [76,77,78,79,80,81,82,83,84,85,86,87,88,89,90,91,92,93,94], including studies of the effects of solvent quality on structural characteristics [95,96,97,98] and self-assembly in various polymer systems [23,79,89]. Herein, we briefly introduce the DPD approach, the details of this approach can be found in [72,73,74]. The beads in DPD represent groups of atoms with the motion of these beads governed by the Newton’s equations of motion [74]. There are three contributions to the pairwise additive force $F_{ij}$ exerted on a bead i by a bead j: conservative ($F_{ij}^{C}$), dissipative ($F_{ij}^{D})$, and random ($F_{ij}^{R}$) force. The total force acting on a bead *i*, $F_{tot}=\underset{j\neq i}{\sum }F_{ij}$, is calculated via summation over all forces acting on this bead from the beads located within the characteristic cutoff distance $r_{c};$ the value of $r_{c}$ effectively defines the intrinsic length scale of the model. We chose the conservative force corresponding to the soft repulsion between the beads, which is the most common choice of the conservative force in DPD, as [74]

(1) $$F_{ij}^{C}=\{\begin{array}{c} a_{ij}\left(1-\frac{r_{ij}}{r_{c}}\right)e_{ij}\\ 0 \end{array}\;\begin{array}{c} \left(r_{ij}<r_{c}\right)\\ \begin{array}{c} \left(r_{ij}\geq r_{c}\right) \end{array} \end{array}$$

where $a_{ij}$ is the repulsion coefficient that defines the magnitude of maximum repulsion between the beads i and j, $r_{ij}=\left|r_{ij}\right|$ is the distance between the centers of masses of these beads, $r_{ij}=r_{i}-r_{j}$, and $e_{ij}=\frac{r_{ij}}{r_{ij}}$. The repulsion coefficient between the same types of beads, $a_{ii}$ is often chosen based on the degree of coarse-graining [74,99], while the repulsion parameter between the beads *i*and *j* representing chemically distinct species can be chosen based on the affinities between the species these beads, represented as [74]

(2) $$a_{ij}=a_{ii}+3.27\chi_{ij}$$

where $\chi_{ij}$ is the Flory–Huggins interaction parameter between the respective species; the above expression holds for the bead number density ρ=3, which is the common choice on the number density in DPD.

The dissipative and random forces are coupled to satisfy the fluctuation-dissipation theorem and are taken as [74] $F_{ij}^{D}=-\gamma \omega^{2}\left(r_{ij}\right)\left(e_{ij}\cdot v_{ij}\right)e_{ij},$ and $F_{ij}^{R}=\sigma \omega \left(r_{ij}\right)\zeta_{ij}\cdot t^{-1/2}e_{ij}$*,* respectively, where γ and σ are the strengths of the dissipative and random forces, $v_{ij}=v_{i}-v_{j}$ is the relative bead velocity, Δt is the simulation time step, and $\zeta_{ij}\;$is a symmetric Gaussian random variable with unit variance and zero mean. We use the most common choice of the weight function [74,99] $\omega \left(r_{ij}\right)=1-\frac{r_{ij}}{r_{c}}$; other choices of the weight function are also permitted [100] within the DPD framework.

The bonded beads are connected by harmonic bonds with the interaction potential $U_{bond}=\frac{K_{bond}}{2}{\left(r_{ij}-r_{b}\right)}^{2}$, where $K_{bond}$ is a spring constant and $r_{b}$ is an equilibrium bond length. To minimize topological crossings of bonded polymer chains, a known limitation of DPD, we utilize a modified segmental repulsive potential (mSRP) formulation [101]. Within the mSRP formulation, an additional repulsive force

(3) $$F_{ij}^{SRP}=b\left(1-d_{ij}/d_{c}^{S}\right)e_{ij}^{S}$$

is introduced between the centers of the bonds if the distance between these centers, $d_{ij}=\left|d_{ij}\right|$ is below the cutoff distance [101] $d_{c}^{S}$; herein, $e_{ij}^{S}=\frac{d_{ij}}{d_{ij}}$.

The reference parameters in our DPD simulations are chosen as following [74,99]: the beads number density is ρ=3, the cutoff distance is $r_{c}=1$, the strengths of the dissipative and random forces are γ = 4.5 and σ = 3.0, respectively, and the temperature and bead mass are set to unity in reduced DPD units. For the bonded interactions, we set [87] $K_{b}=10^{3}\;$and $r_{b}=0.7;$ notably, high spring constant values were used in a number of recent DPD studies [102,103,104]. The parameters defining mSRP interactions are set as [101] b=80 and $d_{c}=0.8$. These mSRP parameters were shown to effectively minimize topology violations in the original publication [101] and in the subsequent studies [105]. For the repulsion parameter between the same type of beads, we choose $a_{ii}$ = 78 in reduced DPD units, this value is calculated to reproduce water compressibility and corresponds to a single bead representing three water molecules [99]. This choice of the degree of coarse-graining allows one to relate the reduced DPD length scale, $r_{c}=1$, to a dimensional value of $r_{c}\;\approx$ 0.65 nm, and the reduced unit of time, τ, to the dimensional value of τ≈ 88 ps [99]. The choice of the repulsion parameters between the beads representing chemically distinct moieties ($a_{ij},\;\mathrm{where}\;i\neq j)\;$is discussed further below. The parameters above and all the simulation values below are provided in reduced DPD units [74,99]. The equations of motion are integrated using the velocity-Verlet algorithm with the time step Δt = 0.01 τ. The simulations were carried out using the Large Scale Atomic Molecular Massively Parallel Simulator (LAMMPS) package [106,107] with mSRP code [101]. The simulation snapshots are visualized using the Visual Molecular Dynamics (VMD) software [108].

**Figure 1:**
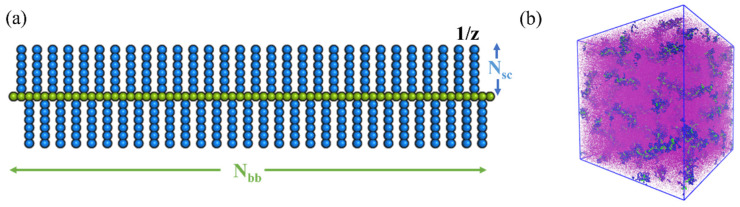
(**a**) Bottlebrush architecture, backbone (bb), and side chain (sc) beads are shown in green and blue, N_bb_ and N_sc_ denote numbers of beads within the backbone and side chain, respectively. (**b**) Initial distribution of n_MBB_ = 77 bottlebrushes equilibrated within the good solvent; solvent beads are shown in purple using reduced bead size.

The bottlebrush architecture in the reference case is shown in Figure 1a, where the beads representing the backbone are shown in green and the beads within the side chains are shown in blue. The number of backbone and side chain beads are denoted as N_bb_ = 62 and N_sc_ = 6, respectively, and the grafting density is set to z = 1. The grafting density equal to one and the ratio between the number of beads within the backbone and within the side chain is chosen based on the experimental data [42]. We model the cases where the backbone beads have high affinity with the solvent beads (a_bb-sol_ = 78) and low affinity with the side chain beads (a_bb-sc_ = 89). The choice of these parameters based on the measured interfacial tensions for the examples of molecular bottlebrushes synthesized in the concurrent experiments is provided in our recent study [42]. In what follows, we vary the affinities between side chain and solvents (a_sc-sol_) within the relatively wide range.

The box size in all the simulations below involving multiple bottlebrushes is chosen as 60 × 60 × 60. The periodic boundary conditions are applied in all directions. The fraction of bottlebrush beads is set at 5% of total beads within the system. For the reference architecture shown in Figure 1a, the bottlebrush consists of $N_{T}=422$ beads. n_MBB_ = 77 bottlebrushes are dispersed within the simulation box of reference size (Figure 1b). In the simulations below, we also vary the bottlebrush architecture keeping the grafting density fixed but varying the degree of polymerization of the side chain (N_sc_ = 10 and N_sc_ = 20); in these series of simulations n_MBB_ is varied correspondingly to keep the fraction of bottlebrushes fixed at 5%. Initially, we set all the repulsion parameters equal to a_ij_ = 78 between all the beads and equilibrate the system for 2 × 10^6^ time steps to ensure that at the initial time for the production runs the bottlebrushes are well dispersed within the box as shown in Figure 1b. For the production runs, we use 3 × 10^6^ time steps. In all the studies involving multiple bottlebrushes, four independent simulations were analyzed. In all the simulations involving single bottlebrushes, the simulation box size is chosen as 30 × 30 × 30 and the last 1200 frames are used for the analysis.

To characterize the conformations of the bottlebrushes dependent upon solvent quality, we calculate the following characteristics for each bottlebrush at a chosen simulation time step *t*: the squared radius of gyration, $R_{g}^{2},$ shape anisotropy, $\kappa^{2}$, and acylindricity, c. The squared radius of gyration of each bottlebrush within the system is calculated as a first invariant of a diagonalized gyration tensor $\hat{S}$ as

(4) $$R_{g}^{2}=I_{1}=tr\;\left[\hat{S}\right]\;=\lambda_{1}+\lambda_{2}+\lambda_{3},\;$$

where $\lambda_{3}\geq \lambda_{2}\geq \lambda_{1}$ are the eigenvalues of the gyration tensor with the components $S_{xy}=\frac{1}{N_{T}^{2}}\Sigma_{i=1}^{N_{T}}\Sigma_{j>i}^{N_{T}}\left(x_{i}-x_{j}\right)\left(y_{i}-y_{j}\right);$ herein the summation is taken over all beads within the bottlebrush, $N_{T}$. A relative shape anisotropy of a bottlebrush is defined via first ($I_{1}$) and second invariants of gyration tensor, $I_{2}=\lambda_{1}\lambda_{2}+\lambda_{2}\lambda_{3}+\lambda_{3}\lambda_{1},$ as [109]

(5) $$\kappa^{2}=1-3\frac{I_{2}}{I_{1}^{2}}$$

Shape anisotropy is used to characterize shapes of objects with complex geometries such as polymers with various architectures [109,110,111,112]. The relative shape anisotropy ranges between $\kappa^{2}=0$ for an object with high symmetry and $\kappa^{2}=1$ for the points located on a line [109]. For a planar object [109] (with $\lambda_{1}=0\;$and $\lambda_{2}=\lambda_{3}$) $\kappa^{2}=0.25$. For linear polymer chains, the shape anisotropy is calculated as $\kappa^{2}\approx 0.43$ in a good solvent and $\kappa^{2}\approx 0.39\;$in the theta solvent [113,114,115]. 

Finally, we also calculate acylindricity

(6) $$c=\lambda_{2}-\lambda_{1}$$

and the ratio between the largest and smallest eigenvalues for each bottlebrush $\lambda_{3}/\lambda_{1}$. 

To analyze all the above structural characteristics upon equilibration, the data are averaged within the last 6 × 10^6^ time steps (using the last 1200 frames) for the case of a single bottlebrush. For the cases of multiple bottlebrushes, the data are averaged over all the bottlebrushes, n_MBB_, (n_MBB_ = 77 in the reference case) within 60 frames (last 3 × 10^5^ time steps) in four independent simulation sets.

## 3. Results and Discussions

### 3.1. Characterizing Conformation of a Single Bottlebrush Dependent upon Solvent Quality

**Figure 2:**
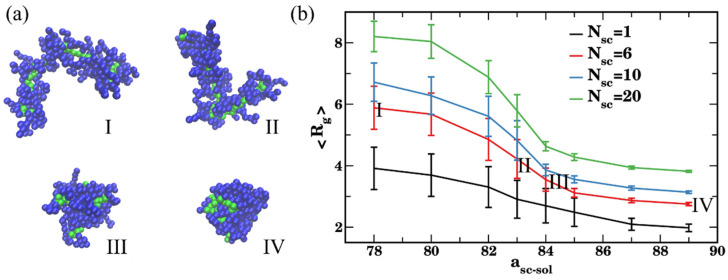
(**a**) Late time snapshots of a bottlebrush conformations (N_sc_ = 6) in solvents of various quality: a_sc-sol_ = 78 in (I), a_sc-sol_ = 83 in (II), a_sc-sol_ = 85 in (III), and a_sc-sol_ = 89 in (IV). (**b**) Radius of gyration of the bottlebrush upon equilibration in solvents of various quality from the good solvent (a_sc-sol_ = 78) to the poor solvent (a_sc-sol_ = 89); black, red, blue, and green curves correspond to N_sc_ = 1, 6, 10, and 20, respectively. The data are averaged over last 6 × 10^6^ steps (1200 frames), error bars represent standard deviations.

We first characterized equilibrium conformation of a single bottlebrush with a chosen architecture dependent upon solvent quality. Specifically, we calculated the radius of gyration, relative shape anisotropy, eigenvalue ratio, and acylindricity of a single bottlebrush (Equations (4)–(6)) at late times upon reaching an equilibrated conformation in solvents of various qualities. These structural characteristics are then compared with the same structural characteristics of bottlebrushes within the agglomerates formed during the self-assembly of multiple bottlebrushes (Section 3.2 below) to identify the effect of agglomeration on these characteristics. We vary the repulsion parameter a_sc-sol_ between the side chain and solvent beads to tailor the side chain affinity to the solvent. The side chain–solvent repulsion parameters are chosen in independent series of simulations as a_sc-sol_ = {78,80,82,83,84,85,87,89}, where a_sc-sol_ = 78 represents a high affinity corresponding to a good solvent and a_sc-sol_ = 89 indicates a low affinity corresponding to a poor solvent. Figure 2a shows representative late time snapshots of a bottlebrush in solvents of different qualities, solvent beads are not shown for clarity of representation. Upon the decrease in the affinity of the side chain and solvent beads, the transition from the random coil to the globule conformation is clearly observed by comparing bottlebrush conformations I–IV in Figure 2a.

The collapse of the bottlebrush upon decreasing the side chains’ solubility in the solvent for various lengths of the side chain N_sc_ = 1, 6, 10, 20 is evident from the decrease in the average radius of gyration upon equilibration for all the N_sc_ considered (Figure 2b). This collapse is observed at conditions close to the theta condition for the linear chains interacting with solvent with a_sc-sol_ = 83 (see Figure S1 of SI). While for the shortest side chain (N_sc_ = 1) this decrease is gradual, the effect becomes more pronounced with an increase in the side chain length, N_sc_. This effect can be attributed to both, an increase in the relative fraction of solvophobic beads at higher N_sc,_ and more pronounced backbone stretching at this high grafting density for the longer side chains. The effect of the backbone stretching is also apparent in Figure S1 of SI, where the average end-to-end distance of the entire bottlebrush, <R_e_>, and an average radius of gyration of the backbone are shown to exhibit the same trends with varying solvent quality. Similar behavior as shown in Figure 2b was previously reported in a coarse-grained MD study [57] of brushes with solvophobic backbones (recall that the backbone in our study is solvophilic), which showed a coil-to-globule transition close to the theta condition for the linear chain upon reducing side chain affinity with more pronounced effect for the longer side chains.

**Figure 3:**
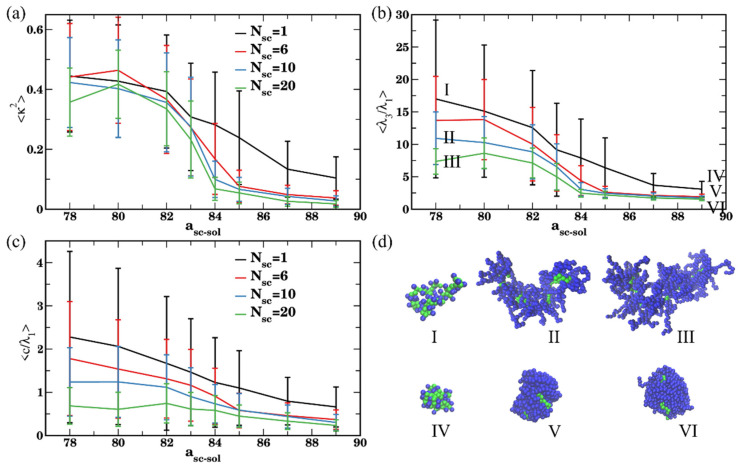
(**a**) Shape anisotropy *κ*^2^, (**b**) ratio of eigenvalues $\lambda_{3}/\lambda_{1}$, and (**c**) acylindricity *c* normalized by the eigenvalue $\lambda_{1}$. The data are averaged over last 6 × 10^6^ steps (1200 frames), error bars represent standard deviations. Black, red, blue, and green curves correspond to N_sc_ = 1, 6, 10, and 20, respectively. (**d**) Late time snapshots for bottlebrushes with N_sc_ = 1 in good and poor solvents (a_sc-sol_ = 78 in (I), a_sc-sol_ = 89 in (IV), respectively), bottlebrushes with N_sc_ = 10 in good and poor solvents (a_sc-sol_ = 78 in (II), a_sc-sol_ = 89 in (V), respectively), and bottlebrushes with N_sc_ = 20 in good and poor solvents (a_sc-sol_ = 78 in (IV) and a_sc-sol_ = 89 in (VI), respectively).

To gain further insights into the conformation of a bottlebrush in solvents of various quality with respect to the side chains, we next characterize the relative shape anisotropy, $\kappa^{2},\;$acylindricity c, and the ratio between the largest and smallest eigenvalues, $\lambda_{3}/\lambda_{1}$, for the same series of simulations (Figure 3). For all the cases considered, the values of $\kappa^{2}$ decrease with the decrease in the side chain affinity to the solvent (Figure 3a). When the side chains are in a good solvent (a_sc-sol_ = 78), for the bottlebrushes with N_sc_ = 1, 6, and 10 the calculated average values of the shape anisotropy $\kappa^{2}$ are close to ≈0.43, the values previously reported for linear chains in a good solvent [113,114] (see the representative snapshot in Figure 3d(I)). For the longest side chain length considered, N_sc_ = 20, the average value of $\kappa^{2}$ is somewhat lower than that for all the shorter side chains. This difference can be attributed to the relatively low ratio between the backbone and the side chain degrees of polymerization (see the snapshot in Figure 3d(V)). Correspondingly, the average value of $\kappa^{2}$ in this case is somewhat closer to that for the star polymer [116]. For the bottlebrushes in poor solvent with respect to the side chains (a_sc-sol_ = 89), the shape anisotropy $\kappa^{2}$ is close to zero for N_sc_ = 6, 10, and 20, indicating formation of highly symmetric globules for all these cases (Figure 3d(II,VI)). For the shortest side chain length of N_sc_ = 1, the average $\kappa^{2}$ is notably higher in poor solvent than that for all the other architectures, but it still remains relatively low ($<\kappa^{2}>\approx \;$0.1). The latter indicates collapse of the bottlebrush into a structure with relatively low symmetry (see the snapshot in Figure 3d(IV)).

Same trends are further confirmed by comparing an average ratio between the largest and smallest eigenvalues, $<\lambda_{3}/\lambda_{1}>$ (Figure 3b), and an average acylindricity normalized on the smallest eigenvalue, $\langle c/\lambda_{1}\rangle$ (Figure 3c). The normalization on the $\lambda_{1}$ in the latter plot is needed for more direct comparison between the structural characteristics of macromolecules with highly disparate dimensions; the acylindricity c, as well as $\lambda_{2}/\lambda_{1}$ ratios are provided in Figure S2. These plots confirm that the bottlebrushes remain highly globular for N_sc_ = 6, 10, and 20 at high a_sc-sol_ while for N_sc_ = 1 both $<\lambda_{3}/\lambda_{1}>$ and $\langle c/\lambda_{1}\rangle$ remain relatively high even at the highest a_sc-sol_ considered. For the solvents with high affinity to the side chains, the values $\langle c/\lambda_{1}\rangle$ decrease with an increase in N_sc_, indicating the lowest acylindricity normalized per smallest linear size in the system for the macromolecules with the longest side chains. We also note large error bars in the calculated values in Figure 3a–c for all the cases except the cases corresponding to nearly globular conformations. These large error bars indicate a broad distribution of macromolecular conformations upon equilibration and are on the same scale as observed in the prior studies of shape anisotropy of various polymers [114,117].

### 3.2. Characterizing Bottlebrush Conformations in Solutions

**Figure 4:**
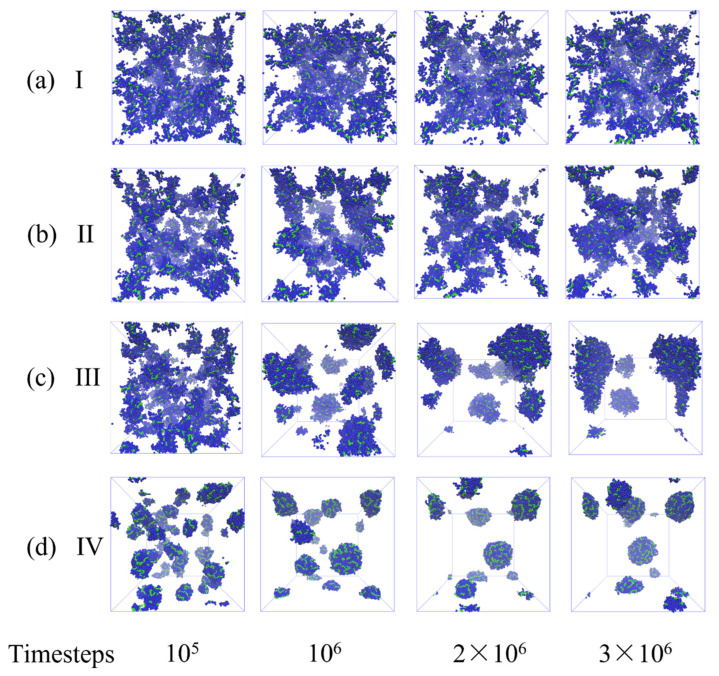
Representative snapshots of time evolution of n_MBB_ = 77 bottlebrushes with N_sc_ = 6 in solvents of various qualities: (**a**) a_sc-sol_ = 78 (Case I), (**b**) a_sc-sol_ = 83 (Case II), (**c**) a_sc-sol_ = 84 (Case III), and (**d**) a_sc-sol_ = 89 (Case IV). The snapshots correspond to the time steps provided below each column, the solvent is not shown for clarity of representation.

In the next series of simulations, we focus on the dynamics of the bottlebrushes during the self-assembly in solvents of various qualities. Initially, the bottlebrushes are well dispersed and equilibrated as detailed in the Methods section. Representative simulation snapshots of time evolution of 5% solution of bottlebrushes (n_MBB_ = 77) in a good solvent (a_sc-sol_ = 78, Case I), in solvents of intermediate quality (a_sc-sol_ = 83 and a_sc-sol_ = 84, Cases II and III, respectively), and in a poor solvent (a_sc-sol_ = 89, case IV) are shown in Figure 4. The bottlebrushes with the side chains with high affinity to the solvent (a_sc-sol_ = 78, Figure 4a) remain well dispersed throughout the entire simulation run. For the intermediate scenarios, the bottlebrushes begin to form clusters but remain mainly dispersed for a_sc-sol_ = 83 (Figure 4b), and are mainly aggregated for a_sc-sol_ = 84 (Figure 4c). In the poor solvents (Figure 4d), the bottlebrushes first form small clusters relatively quickly (see snapshot at t = 10^5^) and then these smaller clusters coalesce forming larger close-to-spherical aggregates (remaining snapshots in Figure 4d).

To characterize the self-assembly of bottlebrushes into aggregates under various conditions quantitatively, we begin with characterizing clusters formation with time for all the above cases. Since our scenarios encompass a range of systems from well-dispersed to agglomerated bottlebrushes, a clear protocol defining a cluster that is suitable for a range of scenarios needs to be identified. The intuitively simple Stillinger criterion [118], which identifies two monomers as belonging to the same cluster if they are separated by a distance smaller than a chosen cutoff distance, is successfully used in a number of studies including studies involving molecular modeling of the self-assembly of bottlebrushes [61,119]. Depending on the system considered, the Stillinger criterion may however strongly overestimate the size of small molecular clusters [120,121,122], counting passing molecules within the given cutoff distance. In the later scenarios, the ten Wolde–Frenkel cluster definition [123], which in addition to the distance criterion accounts for a prescribed number of neighbors, can be utilized [120,121,122]. In DPD simulations, the cutoff distance, which determines whether two beads belong to the same cluster is often chosen [23,124,125] as the model cutoff distance r_c_ defined above. Hence, counting two bottlebrushes as part of the same cluster provided that there is at least a single contact (n_c_ = 1) within the cutoff distance r_c_ between the pair of beads belonging to each bottlebrush is an equivalent of the Stillinger criterion for our system. However, it is reasonable to anticipate that using a single contact would result in strong overestimation of the cluster sizes in the cases when the bottlebrushes are well dispersed or even partially dispersed due to counting short-lived (or passing by) contacts. Hence, we introduce two more criteria for identifying whether two bottlebrushes belong to the same cluster and compare the utility and robustness of all three criteria. 

Since all interactions in DPD are considered only within the model cutoff distance r_c_, an extension of the clustering criteria based on the single contact defined above needs to incorporate a specific required number of neighbors within this distance. The minimum required number of neighbors in turn can be defined based on the coordination number calculated up to the cutoff distance r_c_. The coordination number of a single bead within the entire system calculated using radial distribution function up to the cutoff distance r_c_ is 12.52. Hence, an additional criterion to identify whether two bottlebrushes belong to the same cluster can be introduced by requiring that at least one bead within one bottlebrush is effectively fully surrounded by the beads of another bottlebrush. This criterion corresponds to the minimum number of contacts required to consider both bottlebrushes belonging to the same cluster to be based on the coordination number, namely n_c_ = 13 contacts between the beads belonging to two different bottlebrushes. Finally, we define another, an even more strict criterion that effectively requires all the beads within the single side chain of one bottlebrush to be fully surrounded by the beads of another bottlebrush for these two brushes to belong to the same cluster. This number is based on the coordination number of a single bead multiplied by the degree of the polymerization of the side chain, n_c_ = 13 N_sc_ (or n_c_ = 78 in the reference case scenario for N_sc_ = 6). 

**Figure 5:**
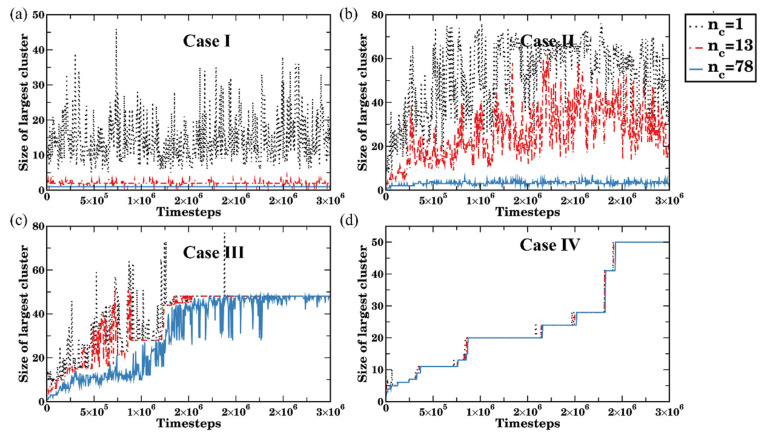
Time evolution of the size of the largest cluster size (number of bottlebrushes) in various solvents: (**a**) a_sc-sol_ = 78 (Case I), (**b**) a_sc-sol_ = 83 (Case II), (**c**) a_sc-sol_ = 84 Case (III), and (**d**) a_sc-sol_ = 89 (Case IV). Black dotted, red dash-dotted, and blue solid lines of the same color represent the number of contacts used to identify clusters (n_c_ = 1, 13, and 78, respectively).

Using all three criteria introduced above (i.e., requiring at least n_c_ = 1, 13, or 13 N_sc_ contacts between the beads of two bottlebrushes for them to belong to the same cluster), we first tracked the distribution of clusters during the time evolution in Cases I–IV. The average size of the largest cluster (the number of the bottlebrushes that belong to this cluster) and the distribution of clusters in selected cases are shown in Figure 5 and Figure S3 respectively. For the well-dispersed Case I (snapshots in Figure 4a), using the single contact cutoff criterion (n_c_ = 1) leads to extensive variations in the size of the largest cluster (from 5 to 46 bottlebrushes, black dotted line in Figure 5a). Hence, as anticipated, this criterion results in significant overestimation of the size of the largest cluster due to counting passing-by brushes as short-lived clusters. With n_c_ = 13 (red dash-dotted line in Figure 5a) and n_c_ = 78 (blue solid line), the calculated sizes of the largest cluster are comparable. While some variations in the largest cluster size are observed with n_c_ = 13 (in some cases counting a small number of transient agglomerates of 2–3 bottlebrushes), for n_c_ = 78 the largest cluster consists of a single bottlebrush during the entire simulation run with no transient agglomerates being accounted for (Figure 5a, black solid line). Similar trends are observed for Case II (Figure 5b); however, the size of the largest cluster correspondingly increases to approximately 25 bottlebrushes on average for n_c_ = 13 (red dash-dotted line). It should be noted that the size of the largest cluster strongly varies with time from 2 to 40 bottlebrushes indicating that the short-lived contacts between the neighboring macromolecules are accounted for while this criterion is used. With n_c_ = 78, the largest cluster size for Case II is around 3 (blue solid line in Figure 5b) indicating a transient nature of visually larger agglomerates (intermediate to late time snapshots in Figure 4b). Similar trends distinguishing various criteria are observed at early times in Case III (Figure 5c), while at late times all the criteria give the same average size of the largest clusters. Finally, the size of the largest cluster increases in a stepwise manner in the Case IV (Figure 5d); notably, all three criteria result in the same largest cluster size. The stepwise largest cluster size increase clearly indicates the non-transient nature of the agglomeration process: namely, if a given bottlebrush joined the largest cluster, it remains a member of this cluster for the entire simulation run. In other words, no contribution to the size of the largest cluster from the transient short-lived aggregates is observed in Case IV.

The distribution of all cluster sizes with time for the time instances corresponding to the snapshots in Figure 4a,d (Case I and Case IV) are shown in Figure S3. These plots further illustrate that while with n_c_ = 1, a large number of transient short-lived agglomerates are accounted for in the good solvent (Figure S3a); with n_c_ = 78, none of these transient agglomerates are accounted for (Figure S3b). On the contrary, for the poor solvent (Case IV) all criteria give essentially identical clusters distribution (Figure S3c,d). 

**Figure 6:**
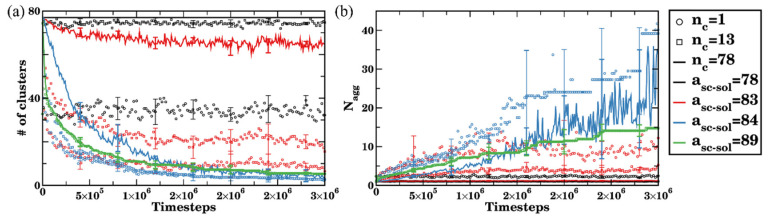
Time evolution of (**a**) number of clusters, and (**b**) aggregation number $N_{agg}$ for a_sc-sol_ = 78 (Case I, in black), a_sc-sol_ = 83 (Case II, in red), a_sc-sol_ = 84 Case (III, in blue), and a_sc-sol_ = 89 (Case IV, in green). Circles, squares, and solid lines of the same color represent the number of contacts used to identify clusters (n_c_ = 1, 13, and 78, respectively). The data are averaged over n_MBB_ = 77 bottlebrushes in four independent simulation sets for each time step, the error bars represent standard deviation.

Next, we calculate an average number of clusters and the aggregation number defined as [23,56]

(7) $$N_{agg}=\sum^{\;}n_{i}P_{i}/\sum^{\;}n_{i},\;$$

where *P_i_* is the size of the cluster *i* (number of bottlebrushes within this cluster) and *n_i_* is the number of clusters of this size; the summation is taken over all the clusters. The time evolution data are summarized in Figure 6 for Cases I–IV (in black, red, blue, and green, respectively) and for all the above cutoff criteria (n_c_ = 1, 13, and 78 are shown by circles, squares, and solid lines of the respective color as marked in the legend). The data points are averaged over four independent simulations, the error bars represent standard deviations. We first focus on the solid curves for all four cases (n_c_ = 78). The dispersed bottlebrushes (Case I) remain well dispersed in all the runs, solid black curves correspond to the number of bottlebrushes, n_MBB_ = 77, (Figure 6a) and respectively $N_{agg}=1\;$(Figure 6b). In other words, using the criteria n_c_ = 78 identifies each bottlebrush as an independent cluster. However, if one uses a single contact cutoff (n_c_ = 1, black circles in Figure 6), the number of clusters is significantly lower and varies extensively with time due to the formation of transient short-lived agglomerates as discussed above for a single simulation run.

Upon agglomeration (Cases II–IV), the bottlebrushes form larger clusters hence the average number of clusters decreases with time (solid red, blue, and green curves in Figure 6a, correspondingly). For Case II, a number of clusters calculated using n_c_ = 78 remains just somewhat lower than the total number of bottlebrushes n_MBB_ (Figure 6a, solid red curve). Using cutoff criteria n_c_ = 1 or 13 and following the time evolution for $N_{agg}$ for Case II (red symbols in Figure 6b) further indicates formation of primarily short-lived agglomerates in this scenario. 

The agglomeration process is distinctly different in Cases III and IV. The process is notably faster for Case IV (green curve, solid line for n_c_ = 78) than that for Case III (in blue, solid line for n_c_ = 78) since the driving force for the agglomeration due to the differences in affinities of the side chains and solvent is the strongest in Case IV. Furthermore, an average aggregation number—which reflects an average size of the cluster—changes approximately stepwise for the average $\;N_{agg}$ (and strictly stepwise for each individual run) for the Case IV, confirming the non-reversibility of the agglomeration process noted above (i.e., a particular bottlebrush joining a cluster belongs to this cluster for the remainder of the simulation run). On the contrary, large absolute values of $N_{agg}$ and extensive variations in these values with time are observed for Case III (blue solid line in Figure 6b), indicating that even using n_c_ = 78 criteria accounts for the notable contribution of short-lived relatively large agglomerates since the transient agglomeration is an inherent feature of the aggregation process for this solvent of intermediate quality. 

**Figure 7:**
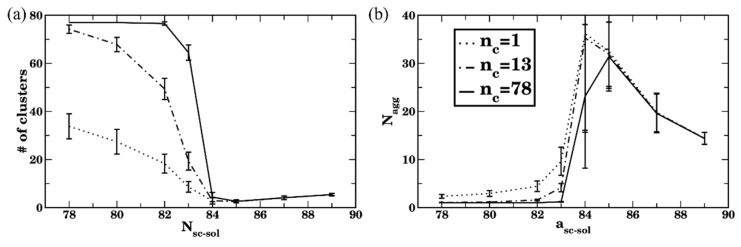
Late time average of (**a**) number of clusters and (**b**) aggregation number for sidechain length N_sc_ = 6. Dotted, dash-dotted, and solid lines of the same color represent the number of contacts used to identify clusters (n_c_ = 1, 13, and 78, respectively). The data are averaged over n_MBB_ = 77 bottlebrushes in four independent simulation sets for each time step, the error bars represent standard deviation.

To identify the applicability and the utility of the chosen criteria, we next analyze an average number of clusters (Figure 7a) and an average aggregation number (Figure 7b) for all three cutoff numbers of contacts as a function of solvent quality, a_sc-sol_. Dotted, dash-dotted, and solid curves correspond to n_c_ = 1, 13, and 78, respectively. These plots clearly show that for the relatively low solvent quality with respect to the side chains, a_sc-sol_ ≥85, all the above cutoff criteria lead to identical results.

For the relatively good solvent quality (78 ≤ a_sc-sol_ ≤ 82), using n_c_ = 1 results in a significant contribution of transient agglomerates into a cluster count, while n_c_ = 13 already allows one to disregard the majority of short-lived transient clusters, with only small differences in measured values of $N_{agg}\;$observed while using n_c_ = 13 or n_c_ = 78 (Figure 7b). Recall that, when using n_c_ = 13 contacts as a cutoff number of contacts for the two bottlebrushes to belong to a single cluster ensures, effectively at least one bead from a chosen bottlebrush is fully surrounded by the beads of another bottlebrush; while using n_c_ =78 contacts ensures that effectively all the beads within the single side chain of one bottlebrush are fully surrounded by the beads of another bottlebrush. Using the criterion n_c_ =78 results in a number of clusters equal to the number of bottlebrushes n_MBB_ in the system (Figure 7a); hence, no passing by bottlebrushes is accounted for if this criterion is used.

For the solvents of intermediate quality (82 ≤ a_sc-sol_ ≤ 85), transient short-lived clusters largely contribute to the dynamics of the self-assembly. Within the transient (short-lived) cluster, a bottlebrush that is at a given time instant belongs to this cluster may migrate away from it and become a member of another cluster. As discussed above, this transient agglomeration is an inherent feature of the aggregation process for the solvents of intermediate quality. Hence, characterizing the agglomeration process using various cutoff criteria provides further insights into the relative contributions of transient aggregation and formation of the long-lived clusters. 

**Figure 8:**
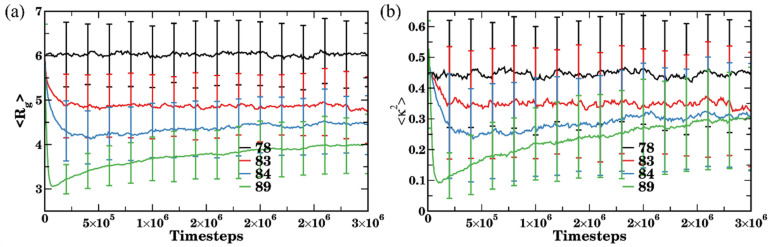
Time evolution of (**a**) radius of gyration and (**b**) shape anisotropy. At each time step, the data are averaged over all n_MBB_ = 77 bottlebrushes in four independent simulation sets, error bars represent standard deviation. Black, red, blue, and green curves correspond to affinities a_sc-sol_ =78, 83, 84, and 89, respectively.

For the same series of the simulations as introduced above (Cases I–IV, Figure 4), we next characterize conformations of bottlebrushes during the assembly process as a function of time by calculating the radius of gyration R_g_ and the shape anisotropy $\kappa^{2}$ (Figure 8). In Case I (black curves), both R_g_ and $\kappa^{2}$ remain approximately constant during all the simulation runs with the values close to those for a single bottlebrush in a good solvent with respect to the side chains. Both these values notably decrease at a relatively early time for Case II (red curves) and maintain these lower values (with respect to Case I) for the remainder of the simulation runs. The equilibrated values of R_g_ and $\kappa^{2}$ in Case II remain close to—but somewhat lower than—the respective values for a single bottlebrush (see direct comparison below).

Unlike the monotonous decrease and saturation of R_g_ and $\kappa^{2}$ in Case II, a shallow dip in both these characteristics is observed in Case III (in blue) and a more pronounced dip is observed in Case IV (in green) at relatively early times prior to the saturation of both characteristics at their equilibrium values. The sharp decrease in both R_g_ and $\kappa^{2}$ in Case IV clearly reflects formation of small nearly globular structures containing only a few bottlebrushes (as evident in Figure 4d at early times). Notably, $\kappa^{2}$ drops to ≈0.1 at early times (in green Figure 8b), while this value is low it is notably higher than $\kappa^{2}$ corresponding to globular structures formed by a single bottlebrush (Figure 3a). An increase in $\kappa^{2}\;$with time up to ≈0.35 and a corresponding increase in R_g_ indicate the conformational changes during the agglomeration process. While the long-lived clusters are formed at late times in both Cases III and IV, our results indicate differences in aggregation processes in these two scenarios. In Case III, a number of short-lived agglomerates are formed and nearly immediately broken-up (as can also be seen from clusters characterization in Figure 6a,b). In other words, relatively higher affinity between the solvent and the side chains in Case III compared to that in Case IV promotes short-lived clusters and hinders formation of long-lived clusters. Furthermore, upon equilibration $\kappa^{2}$ saturates at approximately the same value in Cases III and IV (Figure 8b), indicating similar shapes of the bottlebrushes within the clusters. The average values of R_g_ however remain higher upon equilibration in Case III than that in Case IV, indicating that the bottlebrushes are somewhat more stretched in Case III.

### 3.3. Characterizing Equilibrium Conformations of Bottlebrushes upon Agglomeration

**Figure 9:**
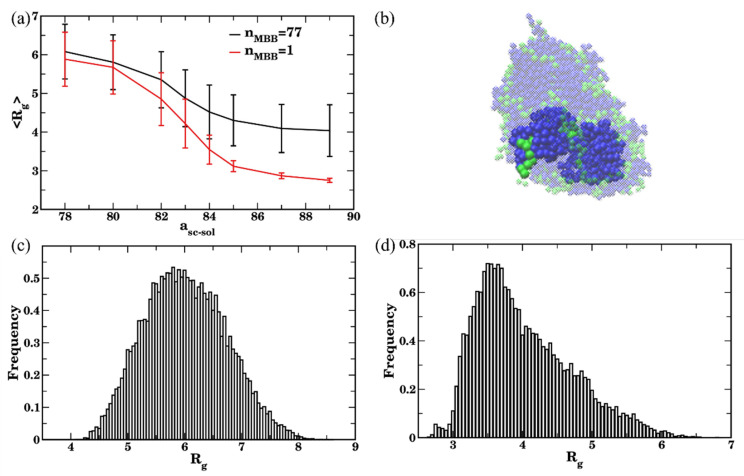
(**a**) Radius of gyration (<R_g_>) of bottlebrushes upon equilibration as a function of sidechains affinity for n_MBB_ = 77 (black curve) and for the single bottlebrush (red curve). The values are averaged over all n_MBB_ = 77 bottlebrushes in four independent simulation sets within last 3 × 10^5^ timesteps (60 frames for each set), error bars represent standard deviation. (**b**) Representative snapshot highlighting a single bottlebrush that is a part of the larger cluster (a_sc-sol_ = 89, t = 10^5^). (**c**,**d**) Distribution of R_g_ in good solvent a_sc-sol_ = 78 (in (**c**)) and in poor solvent a_sc-sol_ = 89 (in (**d**)) for n_MBB_ = 77 bottlebrushes in four sets within the last 3 × 10^5^ timesteps.

We now focus on directly quantifying the effects of the agglomeration of bottlebrushes in various solvents on their equilibrium conformations. We first compare equilibrium radius of gyration of bottlebrushes in solutions containing 5% of bottlebrushes (reference case architecture with N_sc_ = 6 shown in Figure 1a) with that for a single bottlebrush (Figure 9a). As anticipated from the above discussion of the time evolution of structural properties, the value of R_g_ remains the same as that for a single bottlebrush when the bottlebrushes are well-dispersed (a_sc-sol_ = 78, Case I in Figure 4a). With the gradual increase in a_sc-sol_ (a decrease in solvent quality with respect to the side chains), R_g_ decreases similarly to the behavior observed for a single bottlebrush. This decrease is however significantly less pronounced for multiple bottlebrushes (black curve in Figure 9a). The radius of gyration for the multiple bottlebrushes is notably larger than that for a single bottlebrush even for the intermediate solvent quality, for which a large number of transient agglomerates is formed (for example, a_sc-sol_ = 83 in Case II discussed above). Furthermore, an average radius of gyration R_g_ is found to be significantly higher than that for a single bottlebrush in all the cases where the self-assembly into the stable clusters at late times is observed (a_sc-sol_ = [84:89], where a_sc-sol_ = 84 and a_sc-sol_ =89 are the Cases III and IV analyzed above).

It is instructive to characterize the distribution of the values of R_g_ in the case of multiple bottlebrushes. For the dispersed case (a_sc-sol_ = 78), the distribution of R_g_ is symmetric (Figure 9c) with the peak at the average value reported in Figure 9a. For the case of the poor solvent (a_sc-sol_ = 89), the peak is clearly shifted to the lower value (Figure 9d) and is notably lower than the average R_g_ (Figure 9a). The minimum value in this distribution R_g_ ≈ 3.0 corresponds to that observed for a single bottlebrush in the same solvent (i.e., globular configuration of a single bottlebrush), while the highest value at the end of the long tail (R_g_ ≈ 6.2) corresponds to the value for the same chain in a coiled conformation. This asymmetric distribution of the frequencies of occurrences of various values of R_g_ clearly illustrates broad distribution of chain conformations for the chains belonging to bottlebrush aggregates. Specifically, while a large number of bottlebrushes attain conformations close to globular (but remain partially stretched), a notable fraction also remains in close-to-coiled conformations. 

**Figure 10:**
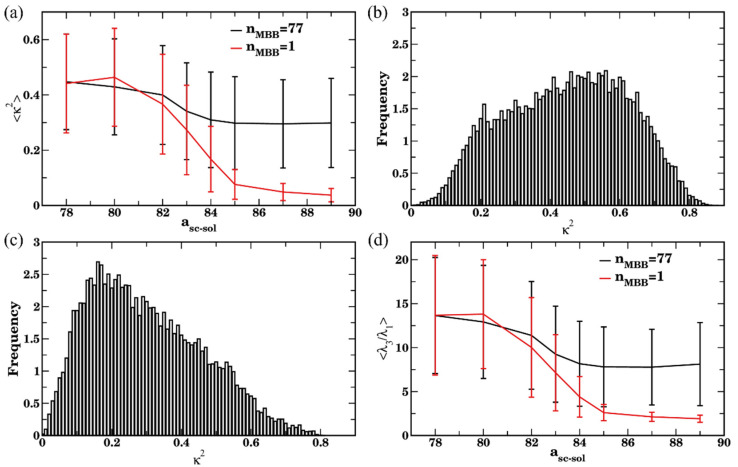
(**a**) Shape anisotropy *κ*^2^ upon equilibration as a function of side chains affinity for n_MBB_ = 77 (black curve) and for the single bottlebrush (red curve). (**b**,**c**) Distribution of the *κ*^2^ in good solvent a_sc-sol_ = 78 (in (**b**)) and in poor solvent a_sc-sol_ = 89 (in (**c**)) for 77 bottlebrushes in four sets with the last 3 × 10^5^ time steps. (**d**) Ratio of eigenvalues λ_3_/λ_1_ upon equilibration as a function of side chains affinity for n_MBB_ = 77 (black curve) and for the single bottlebrush (red curve). In (**a**,**d**), the data are averaged over all 77 bottlebrushes in four sets within the last 3 × 10^5^ time steps (60 frames for each set), error bars represent standard deviation. In (**b**,**c**), data used to plot distributions includes relevant characteristics of all 77 bottlebrushes in four sets within the last 3 × 10^5^ timesteps.

Further details on the configurations of the bottlebrushes in these systems are revealed via analysis of the shape descriptors. The average shape anisotropy, $\;\kappa^{2},$ attains significantly higher values for all the cases corresponding to the formation of relatively large agglomerates than that for a single bottlebrush, as can be seen by comparing red and black curves in Figure 10a for a_sc-sol_ ≥83. These higher values of $\kappa^{2}$ indicate that the shape of a bottlebrush belonging to an agglomerate, on average, remains somewhat closer to that for the polymer chains in a theta-solvent (recall that $\kappa^{2}\approx 0.39\;\mathrm{for}\;\mathrm{the}\;\mathrm{liner}\;\mathrm{chain}\;$in the theta solvent [113,114,115]) than to the chains in globular conformation. For a_sc-sol_ ≥85, a single bottlebrush clearly attains globular conformation (low $\kappa^{2},$ red curve in Figure 10a). Consistent with the distribution of the probability of occurrences of R_g_ (Figure 9d), the probability of occurrences of $\kappa^{2}$ is also broadly distributed and shifted to the left (Figure 10c) for the case of poor solvent with respect to the side chains, while the distribution of $\kappa^{2}$ remains significantly more symmetric, though also broadly distributed for a_sc-sol_ = 78 (Figure 10b). The broad distribution of $\kappa^{2}$ results in the large error bars in Figure 10a. Finally, backbone stretching for the bottlebrushes belonging to the agglomerates is also evident from the relatively high average values of $<\lambda_{3}/\lambda_{1}>$ at a_sc-sol_ ≥85 compared to the same ratio for the single bottlebrushes (compare black and red curves in Figure 10d). Large error bars in Figure 10d at high a_sc-sol_ for the case of multiple bottlebrushes correspond to the broad distribution of their conformations as discussed above (Figure 9d and Figure 10c). To summarize, relating the clustering results using three criteria (Figure 7) and structural characteristics of the bottlebrushes in solutions (with respect to the same characteristics of the single bottlebrush, Figure 9 and Figure 10) show that using n_c_ = 78 allows one to relate bottlebrush conformation in solution for a wide range of solvent qualities to the corresponding agglomeration status. These results also identify a range of solvent qualities (relatively poor solvents) for which any of the criteria may be used to characterize clustering.

### 3.4. Effect of the Side Chain Length on the Agglomeration and Bottlebrush Conformation within Agglomerate

To examine the effects of the bottlebrush architecture on the agglomeration process, multiple bottlebrushes constituting 5% of total volume fractions are placed in a good solvent (Case I, a_sc-sol_ = 78), solvents of intermediate quality (a_sc-sol_ = 83, a_sc-sol_ = 84 for Cases II and III, respectively), and poor solvent (a_sc-sol_ = 89, Case IV). In these series of simulations, the longer side chains are chosen with respect to that in the reference case, specifically N_sc_ = 10 and N_sc_ =20. To keep the total number of bottlebrush beads at 5% of the total number of beads, the numbers of bottlebrushes are reduced with respect to that in the reference case to n_MBB_ = 49 for N_sc_ = 10 (N_T_ = 662) and n_MBB_ = 26 for N_sc_ = 20 (N_T_ = 1262). The representative time evolution simulation snapshots are shown in Figure S4 and Figure S5, respectively. 

We track the time evolution of the number of clusters, aggregation number, radius of gyration R_g_, and shape anisotropy $\kappa^{2}$ to characterize the agglomeration process using the same procedures as introduced above for the reference case architecture (N_sc_ = 6). The trends discussed for the reference bottlebrush architecture are robust and hold for the longer side chains (Figure S6 and Figure S7, respectively). Note that the dip in $\kappa^{2}$ at early times for Case IV is more pronounced for the longer side chains (green curves in Figure S7b,d) than that for the reference bottlebrush architecture. Furthermore, the average shape anisotropy at equilibrium for Case IV (N_sc_ = 10 and 20) and Case III (N_sc_ =10) is also notably lower than that observed in the respective cases for N_sc_ = 6 (Figure S7b,d). This can be attributed to the fact that the clusters containing a smaller number of bottlebrushes are formed if bottlebrushes with longer side chains are used (both at early times and at equilibrium), hence there is effectively smaller relative influence of the neighboring bottlebrushes on the structural characteristics of a chosen bottlebrush. 

**Figure 11:**
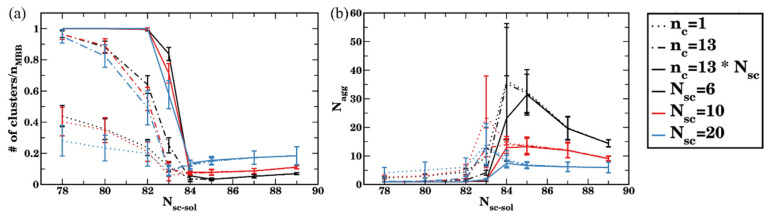
(**a**) Number of clusters scaled by total number of bottlebrushes and (**b**) average aggregation number as a function of solvent quality. Black, red, and blue curves correspond to the side chain length N_sc_ = 6, 10, and 20. Dotted, dash-dotted, and solid lines of the same color represent the minimum cutoff value used to identify clusters (1, 13, and 13 multiplied by N_sc_, respectively).

We next compare the agglomerates at equilibrium by calculating the number of clusters and an average aggregation number for all side chain lengths considered (N_sc_ = 6, 10, and 20). The number of clusters at equilibrium normalized on the number of bottlebrushes, n_MBB_, as a function of the side chain affinities using all three cutoff criteria is provided in Figure 11a; circles, squares, and solid lines of the same color correspond to n_c_ = 1, 13, and n_c_ = 13 N_sc_, respectively. This plot clearly shows that effectively requiring at least one side chain of one bottlebrush to be surrounded by the beads of another bottlebrush for the two brushes to belong to the same cluster (i.e., using the cut off criterion n_c_ = 13 N_sc_) for the cases where the bottlebrushes are relatively well dispersed (a_sc-sol_ ≤ 82) results in the number of clusters equal to the number of bottlebrushes. However, using the cutoff criteria n_c_ = 1 or 13 for the same side chain affinities to the solvent results in a significantly smaller number of clusters for all N_sc_ considered (black, red, and blue symbols and solid lines in Figure 11a for N_sc_ = 6, 10, and 20, respectively). This behavior reflects the relative contribution of formation and breaking of transient short-lived agglomerates as discussed above for the reference case scenario, the same behavior holds for the longer side chains. Note that, for N_sc_ = 6, the same data as discussed above (Figure 7) are used; herein however the data are normalized on n_MBB_ for clarity of comparison between different side chain lengths.

For the simulations of bottlebrush conformations in relatively poor solvents (a_sc-sol_ >84), all three criteria defined above result in an identical number of clusters upon equilibration (Figure 11a). These results show that the number of clusters in equilibrium normalized on n_MBB_ decreases with an increase in N_sc_ in poor solvent. Note that an average size of the cluster in equilibrium expressed via the number of bottlebrush beads—as well as the number of clusters in equilibrium without the normalization on n_MBB_—remain approximately the same for all N_sc_ considered (Figure S8) for the solvents with a_sc-sol_ ≥ 84; this is due to the fact that the same volume fraction (5%) of bottlebrushes with different architectures is used. Finally, the aggregation number—as defined in Equation (7) (expressed in the number of bottlebrushes) with the cutoff criterion n_c_ = 13 N_sc_—is notably lower for higher N_sc_ (for a_sc-sol_ >84) due to the larger number of beads comprising a single bottlebrush. For the solvents with relatively high affinities to the side chains (a_sc-sol_ <82), the lowest cutoff value (n_c_ =1, dotted curves) results in highest $N_{agg}$ for the longest side chain, indicating a more pronounced contribution of short-lived agglomerates, while n_c_ = 13 N_sc_ results in $N_{agg}$ = 1 (Figure 11b). For the solvents of intermediate quality (82 ≤  a_sc-sol_ ≤ 84), the transient clusters are more pronounced for the longer side chains, as can be seen in shifting the transient regions to the left (to the lower a_sc-sol_) in both plots with an increase in N_sc_ (Figure 11).

**Figure 12:**
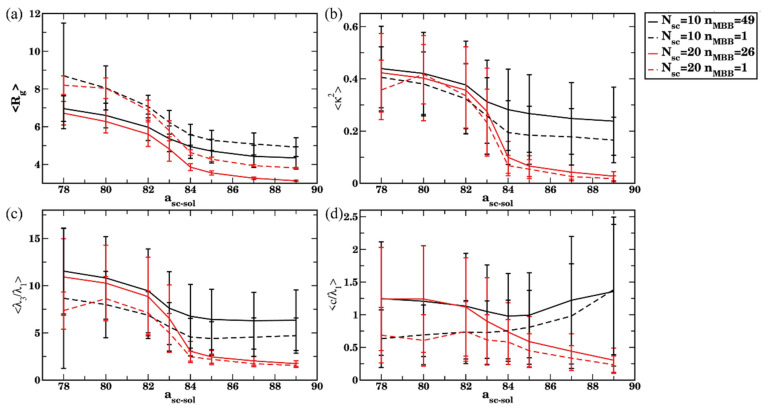
(**a**) Radius of gyration R_g_, (**b**) shape anisotropy *κ*^2^, (**c**) ratio of eigenvalues λ_3_/λ_1_, and (**d**) acylindricity normalized on the smallest eigenvalue, c/λ_1_, of the bottlebrushes as a function of side chain affinities for multiple bottlebrushes (black curves) and single bottlebrushes (red curves). The solid and dash-dotted lines correspond to the sidechain length N_sc_ = 10 and 20, correspondingly. For the multiple bottlebrushes (black curves), the data are averaged over all n_MBB_ bottlebrushes within 60 frames (last 3 × 10^5^ time steps) in four independent simulation runs, error bars represent standard deviations.

Finally, to quantify the effect of clustering on the structural properties of the bottlebrushes with longer side chains, we track structural characteristics of bottlebrushes in solvents of different qualities and compare these characteristics with that for a single bottlebrush. For the solvents with relatively high affinities to the side chains (a_sc-sol_ <82), an average radius of gyration $R_{g}$, average shape anisotropy $\kappa^{2}$, a ratio of eigenvalues $<\lambda_{3}/\lambda_{1}>,$ and an acylindricity normalized by the smallest eigenvalue $\langle c/\lambda_{1}\rangle$ (Figure 12) all remain the same (within the error bars) for the multiple bottlebrushes (black curves, solid for N_sc_ =10 and dashed for N_sc_ =20) as those characteristics for the single bottlebrush (red curves, solid for N_sc_ =10 and dashed for N_sc_ =20). These results clearly indicate that, despite the increased contribution of formation of transient short-lived agglomerates with an increase in a_sc-sol_ within this range, this transient agglomeration does not affect the structural characteristics of the bottlebrushes. A comparison of structural characteristics with the characterization of the agglomeration at equilibrium (Figure 11) clearly indicates that using the cutoff value of n_c_ = 13 N_sc_ allows one to predict the expected trends in bottlebrush conformation based on the calculated average aggregation number. Specifically, this cutoff value primarily captures long-lived clusters, hence it allows one to predict that the structural characteristics of bottlebrushes in solutions will remain the same as those of single bottlebrushes if $N_{agg}=1$. While using lower cut off values (n_c_ =1 or 13) allows one to capture an existence of transient agglomerates at the same range of solvent qualities, these criteria are not sufficient to differentiate short-lived or transient and long-lived clusters.

On the contrary, in relatively poor solvents (a_sc-sol_>84), the average radius of gyration $R_{g}$, average shape anisotropy $\kappa^{2}$, a ratio of eigenvalues $<\lambda_{3}/\lambda_{1}>,$ and an acylindricity normalized by the smallest eigenvalue $\langle c/\lambda_{1}\rangle$ (Figure 12) differ significantly for the multiple bottlebrushes (black curves, solid for N_sc_ = 10 and dashed for) N_sc_ = 20 and the single bottlebrush (red curves, solid for N_sc_ = 10 and dashed for N_sc_ = 20). These trends are the same as observed in the reference case (N_sc_ = 6) and correspond to the relative stretching of the bottlebrushes within the long-lived clusters. The average $\kappa^{2}$ is lowest for N_sc_ = 20 reflecting lower relative influence of the neighboring bottlebrushes on the structural characteristics of a chosen bottlebrush within the clusters since these clusters on average encompass a smaller number of bottlebrushes. (Recall that an average size of the cluster in equilibrium expressed via the number of bottlebrush beads remain approximately the same for all N_sc_ considered for the solvents with a_sc-sol_ ≥ 84 since the same volume fraction (5%) of bottlebrushes with different architectures is used.) Our results show that, in relatively poor solvents, all three criteria for the critical number of contacts can be used to describe the clustering process and predict an effect of clustering on the structural characteristics. For the solvents of intermediate quality (82 ≤ a_sc-sol_ ≤84), the transient clusters are more pronounced for the longer side chains as discussed above. Using the highest cutoff n_c_ = 13 N_sc_ allows one to primarily focus on the effects of long-lived clusters, while using smaller cutoff allows one to also account for contribution of transient clustering processes. To summarize, these studies show that the cutoff n_c_ = 13 N_sc_ can be used to predict the anticipated changes in structural characteristics of bottlebrushes upon agglomeration with respect to the same characteristics for the single bottlebrushes for all the solvent qualities considered herein. Finally, we replot the data shown in Figure 11 and Figure 12 parametrically for the average shape anisotropy $\kappa^{2}$ as a function of the aggregation number, $N_{agg}$ (Figure S9). Herein, we only consider $N_{agg}\;$calculated using highest critical number of contacts, n_c_ = 13 N_sc_. A consistent trend showing a decrease in $\kappa^{2}$ with the decrease in $N_{agg}\;$is seen in Figure S9 for all the relatively poor solvent cases. 

## 4. Conclusions

We characterized agglomeration of molecular bottlebrushes by tracking the time evolution of the number of clusters, the size of the largest cluster, and an average aggregation number. We systematically analyzed three different cutoff criteria to identify whether two bottlebrushes belong to the same cluster. This analysis allows one to introduce a clear protocol to identify both short-lived (or transient) and long-lived agglomerates. Our results demonstrate that the cutoff criterion which depends on both the coordination number and the length of the side chain allows one to largely disregard transient or short-lived clusters. Furthermore, this criterion allows one to correlate the agglomeration status with the structural characteristics of bottlebrushes. We characterized conformational changes of the bottlebrush within the agglomerates with respect to that of an isolated bottlebrush in the same solvent. Specifically, we tracked the radius of gyration, shape anisotropy, acylindricity, and the ratios of eigenvalues of the gyration tensor. These studies allowed us to clearly identify a range of solvent qualities at which conformational changes remain negligible; we also showed that only transient short-lived agglomerates are formed in these scenarios. Furthermore, we identified a range of affinities at which distinct differences in conformations of bottlebrushes within the agglomerates and that of an isolated bottlebrush in the same solvent are observed. Specifically, our results demonstrated a distinct increase in both the radius of gyration and the shape anisotropy for the bottlebrush within the agglomerate, which in turn indicates stretching of a bottlebrush within the agglomerates. The characterization of bottlebrush conformations within the agglomerates is an important step in characterizing the relationship between the bottlebrush architecture and material properties. A comparison between the three distinct cutoff criteria to identify whether two bottlebrushes belong to the same cluster introduces a protocol to identify both short-lived transient and long-lived agglomerates and could be further adapted to characterize the agglomeration process of macromolecules with various complex architectures. 

## Data Availability

The data presented in this study are available on request from the corresponding author.

## Supplementary Materials

The following supporting information can be downloaded at: https://www.mdpi.com/article/10.3390/polym14122339/s1, Figure S1: End-to-end distance (<R_e_>) and Radius of gyration (<R_g_>) of the backbone of a bottlebrush; Figure S2: Acylindricity *c* and ratio of eigenvalues $\lambda_{2}/\lambda_{1}$ of a bottlebrush; Figure S3: Time evolution of distribution of clusters in case I and case IV; Figure S4: Representative snapshots of time evolution of n_MBB_ = 49 bottlebrushes with N_sc_ = 10 in solvents of various qualities; Figure S5: Representative snapshots of time evolution of n_MBB_ = 26 bottlebrushes with N_sc_ = 20 in solvents of various qualities; Figure S6: Time evolution of number of clusters and aggregation number ($N_{agg}$) for the side chain length N_sc_ = 10/20; Figure S7: Time evolution of radius of gyration and shape anisotropy for sidechain length N_sc_ = 10/20; Figure S8: Number of clusters and an average size of cluster in number of beads as a function of side chain affinity for N_sc_ = 10 and 20; Figure S9: Shape anisotropy *κ*^2^ as a function of average aggregation number and snapshots of bottlebrush cluster.
