# Supplementary material for: Mesoscale Modeling of Agglomeration of Molecular Bottlebrushes: Focus on Conformations and Clustering Criteria
Source: Polymers (Basel). 2022 Jun 9;14(12):2339. doi: 10.3390/polym14122339 (PMC9227207; doi:10.3390/polym14122339)
Supplement: Supplementary file 1 [file polymers-14-02339-s001.zip › polymers-1733393-supplementary.pdf]

Supporting Information

Mesoscale Modeling of Agglomeration of Molecular Bottlebrushes: Focus on Conformations and Clustering Criteria

Sidong Tu[1], Chandan K. Choudhury[1,2], Michaela Giltner[1,3], Igor Luzinov[1], Olga Kuksenok[1,*]

[1] Department of Materials Science and Engineering, Clemson University, Clemson, South Carolina 29634, United States; sidongt@clemson.edu (S.T.); chandan.k.choudhury@gmail.com (C.K.C.); luzinov@clemson.edu (I.L.), Michaela.giltner@slu.edu (M.G.); okuksen@clemson.edu (O.K.)
[2] Current address: Prescience Insilico Pvt. Ltd., Bengaluru, Karnataka 560037
India
[3] Current address: Oakwood Labs, Oakwood Village, Ohio 44146, United States;

* Correspondence: okuksen@clemson.edu (O.K.)

[1] Department of Materials Science and Engineering, Clemson University, Clemson, South Carolina 29634, United States; sidongt@clemson.edu (S.T.); chandan.k.choudhury@gmail.com (C.K.C.); luzinov@clemson.edu (I.L.), Michaela.giltner@slu.edu (M.G.)
* Correspondence: okuksen@clemson.edu

Supplementary figures

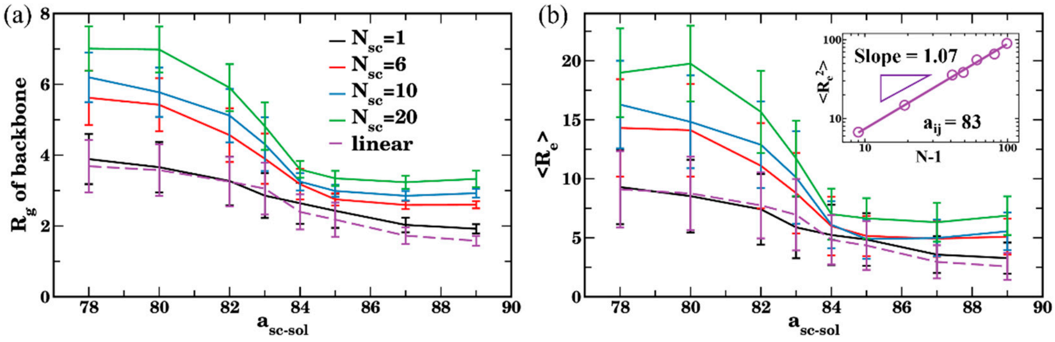

Figure S1. (a) Radius of gyration ($<R_g>$) of the backbone of a bottlebrush only and (b) end-to-end distance ($<R_e>$) upon equilibration in solvents of various qualities. The solvent quality is varied from the good solvent ($a_{sc\text{-}sol}$ = 78) to the poor solvent ($a_{sc\text{-}sol}$ = 89); black, red, blue and green solid lines correspond to N_{sc} = 1,6,10, and 20, respectively. Purple dashed lines corresponds to a linear chain with the degree of polymerization the same as bottlebrush backbone encompassing the same beads as the side chain. All the data points for bottlebrushes are averaged within the last 6×10^6 time steps (1200 frames) and all the data points for linear chains (purple curves) are averaged within the last 1×10^6 time steps (1000 frames), error bars represent standard deviation. The inset in (b) shows the dependence of the mean end-to-end distance on the chain length N, $< R_e^2 > \propto (N-1)^{2\nu}$ for the single linear chain interacting with the solvent with $a_{sc\text{-}sol}$=83; the best fit to the scaling exponent $2\nu = 1.07 \pm 0.028$ is close to the scaling exponent in theta conditions. The data points used in the inset were obtained from independent simulations of the isolated linear chains.

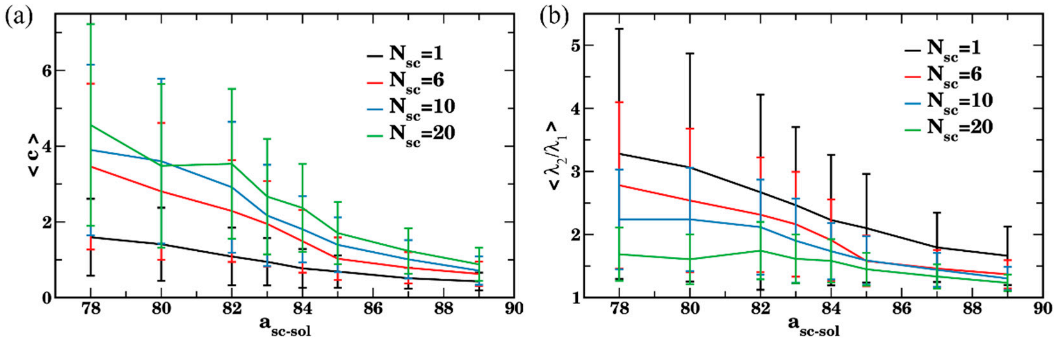

Figure S2. (a) Acylindricity c and **(b)** ratio of eigenvalues λ_2/λ_1 of a bottlebrush. The data is averaged over last 1200 frames, error bars represent standard deviation. Black, red, blue, and green curves correspond to N_{sc} = 1,6,10, and 20, respectively.

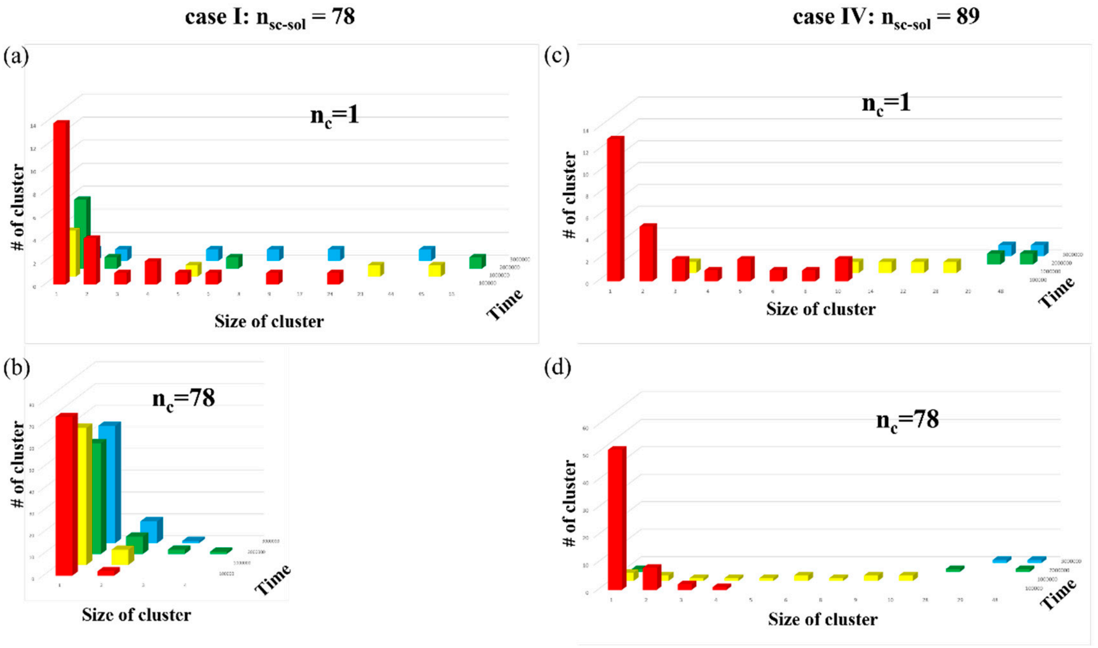

Figure S3. Time evolution of distribution of clusters in **(a-b)** Case I, $a_{sc\text{-}sol}$ =78 and in **(c-d)** Case IV, $a_{sc\text{-}sol}$ =89 for the bottlebrushes with reference architecture (Fig. 1a). Red, yellow, green and blue columns show the clusters distribution at time steps = 10^5, 10^6, 2×10^6 and 3×10^6, corresponding to the two simulation runs with the snapshots provided in Figure 4a and 4d.

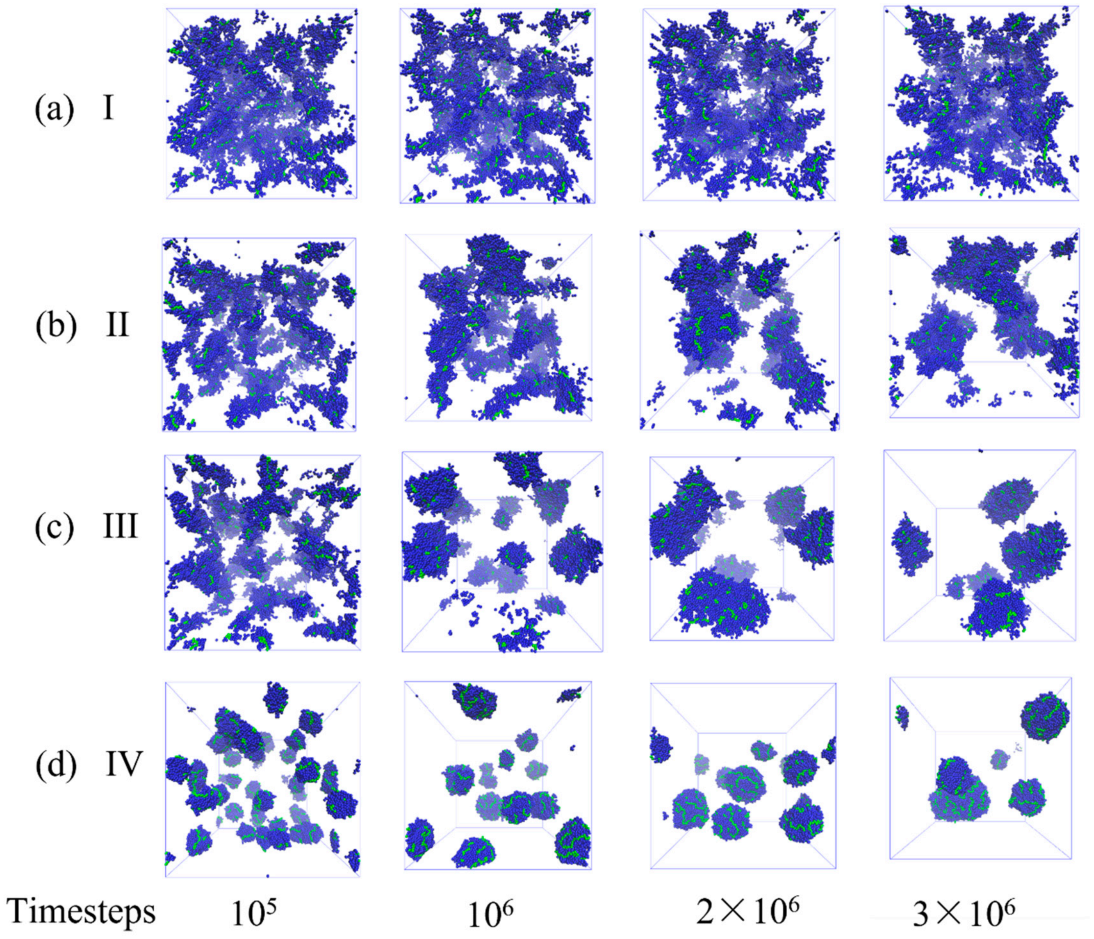

Figure S4 Representative snapshots of time evolution of n_{MBB}=49 bottlebrushes with N_{sc} = 10 in solvents of various qualities **(a)** $a_{sc\text{-}sol}$ = 78, **(b)** $a_{sc\text{-}sol}$ = 83, **(c)** $a_{sc\text{-}sol}$ = 84, and **(d)** $a_{sc\text{-}sol}$ = 89. The snapshots correspond to the time steps provided below each column, the solvent is not shown for clarity of representation.

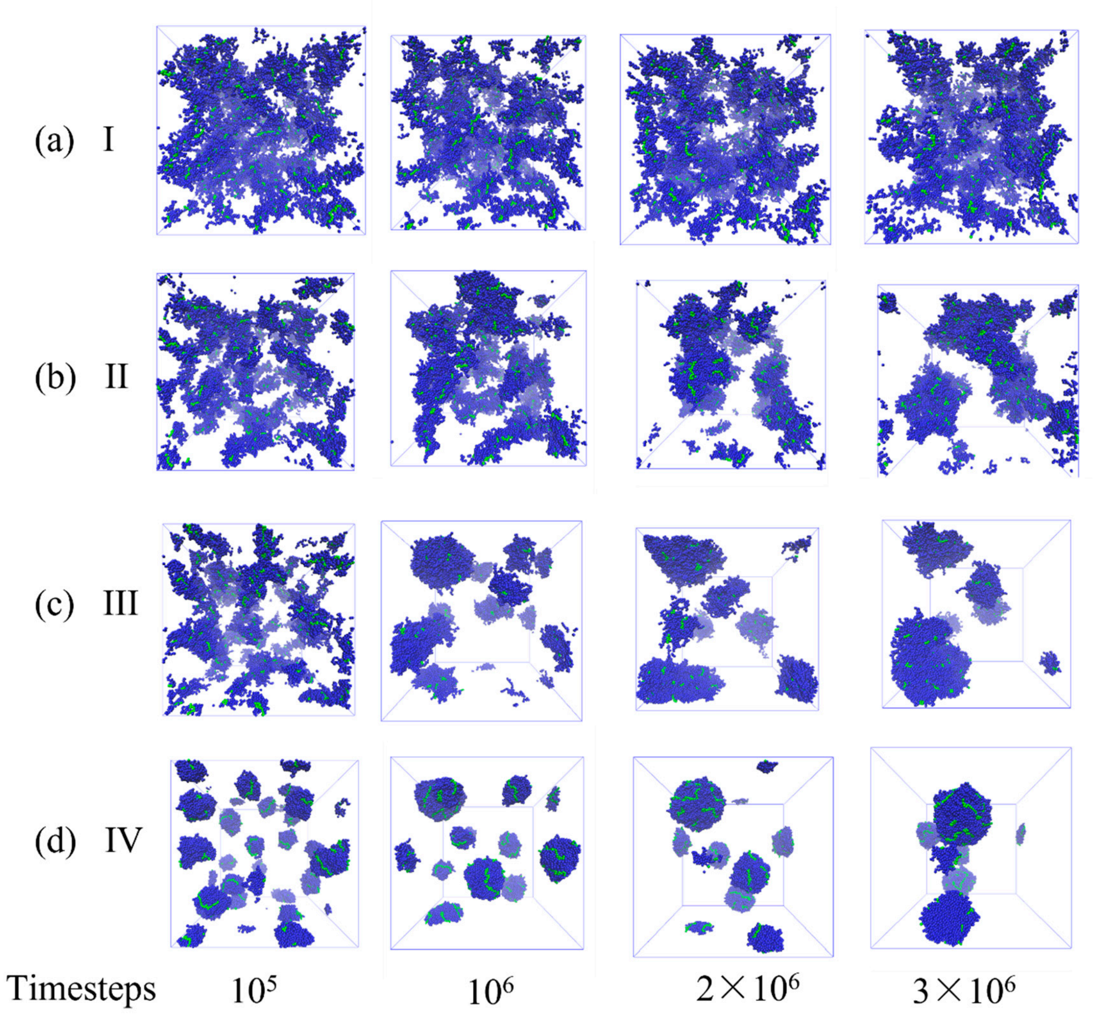

Figure S5. Representative snapshots of time evolution of n_{MBB}=26 bottlebrushes with N_{sc} = 20 in solvents of various qualities **(a)** $a_{sc\text{-}sol}$ = 78, **(b)** $a_{sc\text{-}sol}$ = 83, **(c)** $a_{sc\text{-}sol}$ = 84, and **(d)** $a_{sc\text{-}sol}$ = 89. The snapshots correspond to the time steps provided below each column, the solvent is not shown for clarity of representation.

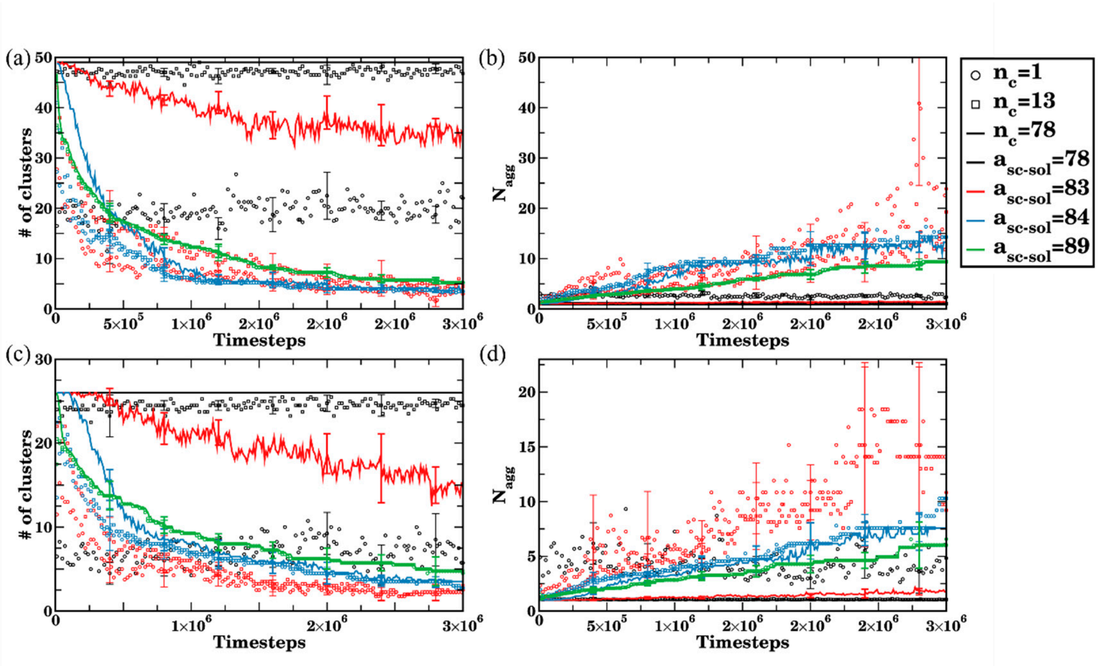

Figure S6. Time evolution of **(a)** number of clusters and **(b)** aggregation number (N_{agg}) for the side chain length N_{sc} = 10. Time evolution of **(c)** number of clusters and **(d)** N_{agg} for the side chain length N_{sc} = 20. Black, red, blue, and green curves correspond to affinities $a_{sc\text{-}sol}$ =78, 83, 84 and 89, respectively. Circles, squares, and solid lines of the same color represent the number of contacts used to identify clusters (n_c=1, 13, and 78, respectively).

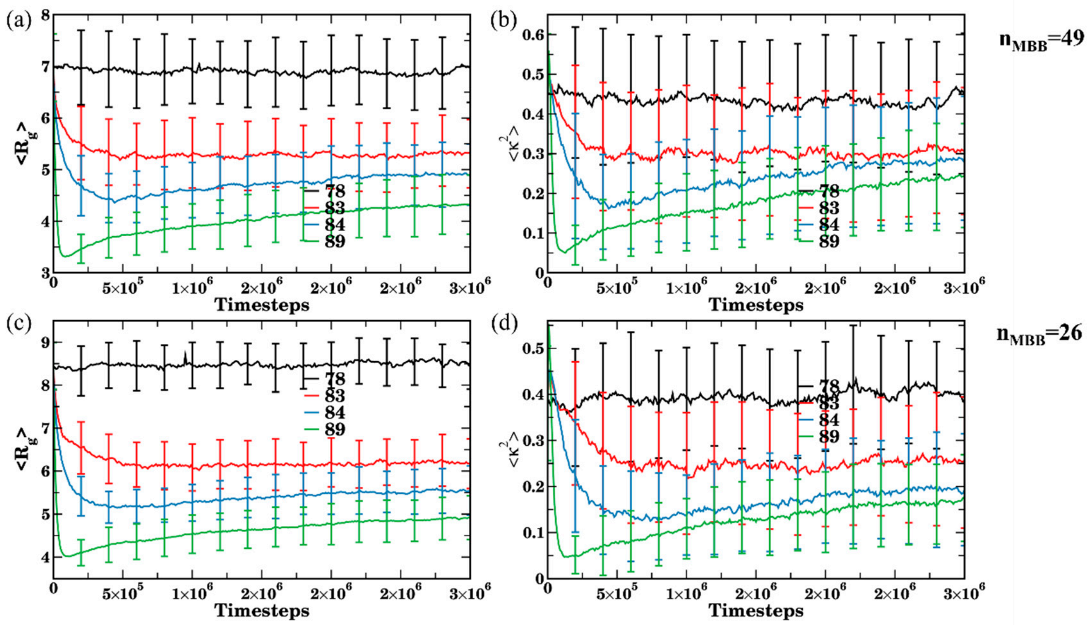

Figure S7. Time evolution of **(a)** radius of gyration and **(b)** shape anisotropy for sidechain length N_{sc}=10 and **(c)** radius of gyration and **(d)** shape anisotropy for sidechain length N_{sc}=20. Black, red, blue, and green curves correspond to affinities $a_{sc\text{-}sol}$ =78, 83, 84 and 89, respectively.

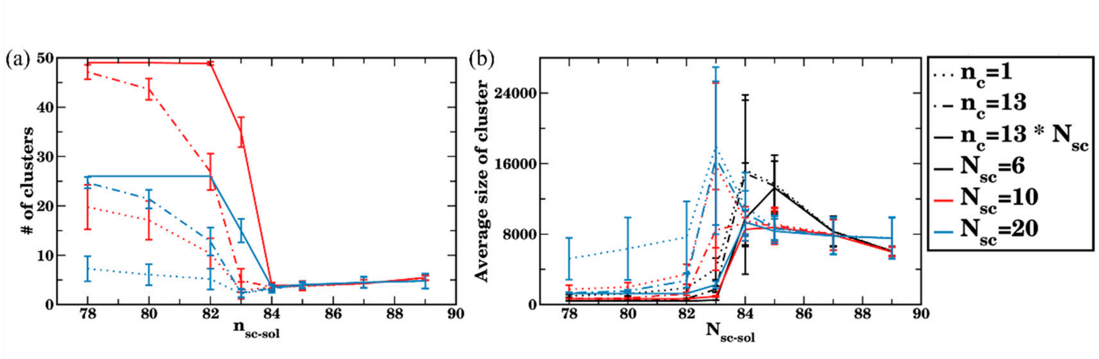

Figure S8. (a) Number of clusters and **(b)** an average size of cluster in number of beads as a function of side chain affinity for N_{sc}=10 and 20. Dotted, dashed-dotted, and solid lines of the same color represent the number of contacts used to identify clusters (n_c=1, 13, and 78, respectively). Black, red and blue curves corresponds to N_{sc} = 6, 10 and 20.

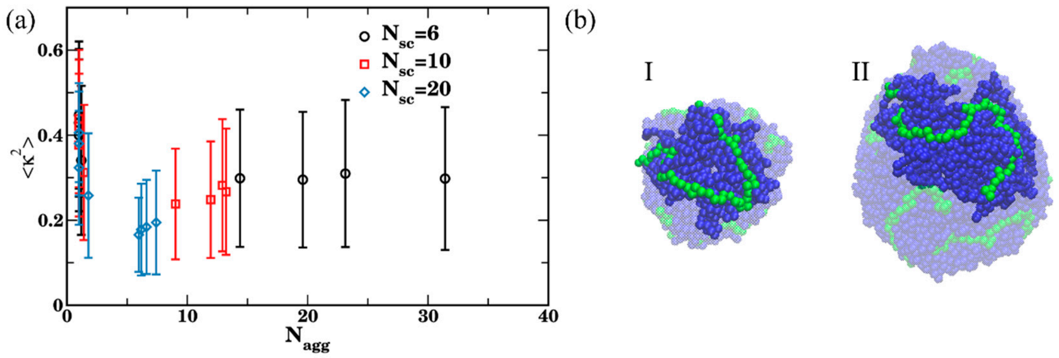

Figure S9. (a) Shape anisotropy κ^2 as a function of average aggregation number (calculated using $n_c = 13N_{sc}$) and **(b)** snapshots of bottlebrush cluster at late time with side chain length N_{sc}=10 (in I) and N_{sc}=20 (in II).
